# The false cleanerfish relies on aggressive mimicry to bite fish fins when benthic foods are scarce in their local habitat

**DOI:** 10.1038/s41598-020-65304-6

**Published:** 2020-05-26

**Authors:** Misaki Fujisawa, Yoichi Sakai, Tetsuo Kuwamura

**Affiliations:** 1 Graduate School of Biosphere Science, Hiroshima University, Higashi-Hiroshima, Japan; 2 Present Address: Fisheries and Marine Technology Center, Hiroshima Prefectural Technology Research Institute, Kure, Hiroshima, Japan; 3 Graduate School of Integrated Sciences for Life, Hiroshima University, Higashi-Hiroshima, Japan; 4 School of International Liberal Studies, Chukyo University, Nagoya, Japan; 5 Present Address: Faculty of Liberal Arts and Sciences, Chukyo University, Nagoya, Japan

**Keywords:** Behavioural ecology, Marine biology

## Abstract

The false cleanerfish, *Aspidontus taeniatus* (Blenniidae), is known for its morphological resemblance to the bluestreak cleaner wrasse *Labroides dimidiatus* (Labridae). It has been suggested that *A. taeniatus*, which acts as a mimic, can easily bite the fins of other fishes that are deceived into requesting cleaning from it or allowing it to approach them. In fact, *A. taeniatus* frequently utilises benthic food items, such as damselfish eggs, the Christmas tree worm *Spirobranchus giganteus*, and the boring clam *Tridacna crocea*. Although geographical variation in the reliance on aggressive mimicry (fin biting) has been reported, the factors have not been determined. We hypothesised that one of the factors is the abundance of benthic food items. To examine our hypothesis, we compared the feeding behaviour of *A. taeniatus* at two locations showing contrasting abundances of benthic food items in Okinawa, southern Japan. The frequency of fin biting by the small *A. taeniatus* in Ishigaki Island, where *S. giganteus* and *T. crocea* were very rare, was significantly higher than that in Sesoko Island, where the two food items were abundant. We conclude that the importance of aggressive mimicry in *A. taeniatus* varies depending on local food conditions.

## Introduction

Aggressive mimicry is a form of imitation in which a predator or parasite (mimic) closely copies another organism (model) that is attractive or harmless to a third organism (dupe) to gain enhanced access to prey. It is widespread among a large breadth of animal groups^1^: e.g. siphonophores^2^, spiders^3,4^, insects^5–8^, snakes^9,10^, birds^11^, and fishes^12–14^. Protective mimicry (Batesian mimicry), in which a prey species gains protection from predators, is also involved in some instances of aggressive mimicry^4,15,16^.

The two functions of mimicry, i.e. protective and aggressive, have been suggested in the case of the false cleanerfish, *Aspidontus taeniatus* (Blenniidae), which resembles the bluestreak cleaner wrasse, *Labroides dimidiatus* (Labridae)^1,17,18^. This is the best-known example of mimicry among coral reef fishes, where cleaning symbiosis is common and widespread^19^. Wickler^1^ emphasised the function of aggressive mimicry mainly through aquarium observation; *A. taeniatus* can bite pieces of fin from fishes that are deceived into requesting cleaning from it or allowing it to approach them. However, from initial quantitative field observations and stomach contents analyses on a coral reef in Okinawa, southern Japan, by Kuwamura^17^, it was determined that *A. taeniatus* feeds primarily on the plumes (tentacles) of tubeworms, *Spirobranchus giganteus* and *Sabellastarte indica* (Polychaeta), occasionally raids the nests of damselfish to eat their demersal eggs, and rarely bites fish fins. Other than fin biting, aggressive mimicry is unlikely to be involved in *A. taeniatus* feeding behaviours, and such a diversity of food items is not reported in the examples of aggressive mimicry mentioned above; other mimic animals almost always use aggressive mimicry when they feed^5,15^. Moreover, comparing its food items and feeding behaviours with those of a congeneric non-mimicking blenny, *Aspidontus dussumieri*, which mainly feeds on filamentous algae and occasionally on *S. giganteus* but never bites fish fins, Kuwamura^17^ concluded that the main function of the similarity of *A. taeniatus* to *L. dimidiatus* is protective mimicry.

Recently, geographical variation in the feeding behaviour of *A. taeniatus* has been reported^20^. On the Great Barrier Reef, in Indonesia and in the Red Sea, it rarely bit fish fins; it relied on other foods, such as damselfish eggs and tubeworms, as observed in Okinawa^17^. However, in French Polynesia, *A. taeniatus* frequently bit fins. Cheney *et al*.^20^ concluded that the relative importance of aggressive mimicry varies between locations, although the factors of geographical variation could not be specified and remain to be identified. They also suggested that the relative importance of mimicry types might vary between life history stages, since fin biting was frequently observed in juveniles (*N* = 2) in Indonesia^20^.

Age-related variation in mimicry has been documented in the field observations of Fujisawa *et al*.^21^ at the same study site (Sesoko Island, Okinawa) that Kuwamura made his initial observations^17^]: *A. taeniatus* utilises aggressive mimicry only when it is small. The frequency of fin biting decreased with its growth, and in turn, egg eating increased. In contrast, it utilised the plumes of *S. giganteus* and the mantles of the boring clam, *Tridacna crocea* (Bivalvia), regardless of its body size. The abundance of benthic materials, such as *S. giganteus* and *T. crocea*, may vary among locations, and may affect the frequency of fin biting by *A. taeniatus*, as Cheney *et al*.^20^ suggested.

Here, we hypothesise that limited benthic food items in a local habitat should affect the reliance on aggressive mimicry in the feeding behaviour of *A. taeniatus*, in addition to its life history stages. To test the hypothesis, we conducted field observations on the feeding behaviour of *A. taeniatus* on the fringing reefs of Ishigaki Island, Okinawa, approximately 400 km southwest of Sesoko Island. Ishigaki reefs seldom harboured the benthic food items, *S. giganteus* and *T. crocea*, preferred by *A. taeniatus*, in contrast to the Sesoko reefs, where these are plentiful. By comparing the data obtained at Ishigaki Island with those from Sesoko Island^21^, we examined the differences in the reliance on fin biting in the feeding behaviours of *A. taeniatus* and discussed the factors promoting geographical variation with regard to conditional feeding tactics.

## Results

We conducted behavioural observations on eight individuals of *A. taeniatus* in Ishigaki Island (Supplementary Table S1) and compared with those of 40 individuals in Sesoko Island (Table S2).

### Abundance of food items

*A. taeniatus* utilised four types of food items on Ishigaki Island, as on Sesoko Island^21^: the tentacles of *S. giganteus*, the mantle edge of *T. crocea*, the fins of other fishes, and the demersal eggs of damselfishes.

**Figure 1:**
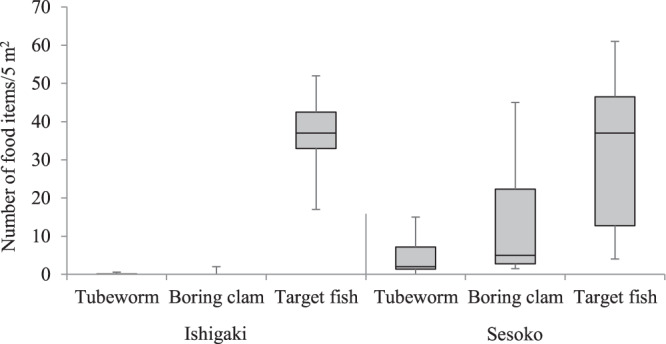
Comparison of the density of food items between Ishigaki Island (left) and Sesoko Island (right). Upper bars represent maximum, lower bars minimum, and middle bars median. For the benthic food items in the figure: Tubeworm, *Spirobranchus giganteus*; Boring clam, *Tridacna crocea*.

The density of fishes (per 5 m^2^), e.g., small damselfishes *Chrysiptera cyanea* and *Pomacentrus moluccensis* (Pomacentridae), targeted for fin biting was not significantly different between Ishigaki Island (*N*_1_) and Sesoko Island (*N*_2_) (medians = 37 and 37, ranges = 17–52 and 4–61, respectively; *N*_1_ = 7, *N*_2_ = 7; Mann–Whitney *U* test: *U* = 22.5, *P* = 0.59; Fig. 1). Two benthic food items, *S. giganteus* and *T. crocea* were very rare on Ishigaki Island (Fig. 1); the total number (per 5 m^2^) was significantly less than that on Sesoko Island (medians = 0 and 16, ranges = 0–2 and 2.3–45, respectively; *N*_1_ = 7, *N*_2_ = 7; Mann–Whitney *U* test: *U* = 0, *P* = 0.002). Although we have no quantitative data on fish eggs, large damselfish adults such as *Abudefduf sexfasciatus*, which were abundant on Sesoko Island^21^, were rare on Ishigaki Island.

### Relationship between body size and feeding frequency

**Figure 2:**
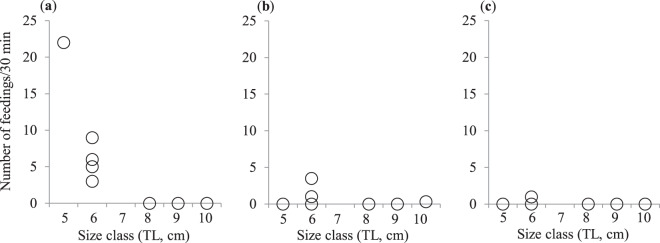
Relationship between the body size of the false cleanerfish *Aspidontus taeniatus* and its feeding frequency on fish fins (**a**), the Christmas tree worm *Spirobranchus giganteus* (**b**), and the boring clam *Tridacna crocea* (**c**) on Ishigaki Island.

The frequency of fish-fin biting was significantly and negatively correlated with the body size of *A. taeniatus* on Ishigaki Island (Spearman’s rank correlation, *r*_*s*_ = −0.91, *N* = 8, *P* = 0.016; Fig. 2a), as on Sesoko Island^21^. We could not analyse the correlation between body size and the frequency of egg eating, because egg eating was only observed once on Ishigaki Island (an *A. taeniatus* individual of 10 cm total length [TL] solely fed on the eggs of a damselfish, *Pomacentrus chrysurus*, of 6 cm TL). Large *A. taeniatus* often formed a group to raid the nests of larger damselfish on Sesoko Island^21^, and such a conspecific aggregation of five *A. taeniatus* was also observed on Ishigaki Island, although the actual raiding of damselfish nests (i.e. egg predation) was not seen. The feeding frequencies on *S. giganteus* and *T. crocea* on Ishigaki Island were not significantly correlated with the body size of *A. taeniatus* (Spearman’s rank correlation, *r*_*s*_ = 0.044 and –0.176, respectively; *N* = 8, *P* = 0.91 and 0.64 each; Fig. 2b,c), as on Sesoko Island^21^.

### Geographical variation in feeding frequency

**Figure 3:**
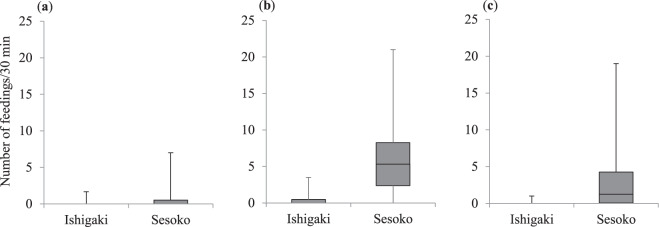
Comparison of the feeding frequency on fish eggs (**a**), the Christmas tree worm *Spirobranchus giganteus* (**b**), and the boring clam *Tridacna crocea* (**c**) by the false cleanerfish *Aspidontus taeniatus* between Ishigaki Island and Sesoko Island. Upper bars represent maximum, lower bars minimum, and middle bars median.

The frequency of egg eating (per 30 min) on Ishigaki Island (*N*_1_) was not significantly different from that on Sesoko Island (*N*_2_) (medians = 0 and 0, ranges = 0–1.7 and 0–7, respectively; *N*_1_ = 8, *N*_2_ = 40; Mann–Whitney *U* test: *U* = 130, *P* = 0.29; Fig. 3a). The feeding frequencies on *S. giganteus* and *T. crocea* on Ishigaki Island were significantly lower than those on Sesoko Island (*S. giganteus*, medians = 0 and 5.3, ranges = 0–3.5 and 0–21, respectively; Mann–Whitney *U* test: *U* = 34, *P* = 0.0005; Fig. 3b; and *T. crocea*, medians = 0 and 1.25, ranges = 0–1 and 0–19, respectively; *U* = 53.5, *P* = 0.003; Fig. 3c).

**Figure 4:**
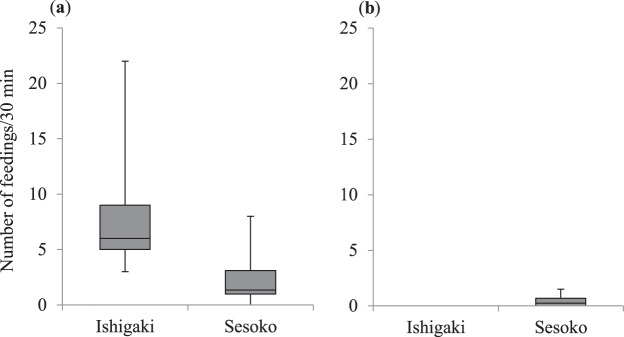
Comparison of the frequency of fish-fin biting by the false cleanerfish *Aspidontus taeniatus* between Ishigaki Island and Sesoko Island. (**a**) Small *A. taeniatus* (<7 cm total length [TL]), (**b**) large *A. taeniatus* (≥ 7 cm TL). Upper bars represent maximum, lower bars minimum, and middle bars median.

Since the frequency of fish-fin biting was negatively correlated with body size of *A. taeniatus*^21^ (Fig. 2a), we made a comparison between the two study sites for the small size class (<7 cm TL) and large size class (≥7 cm TL) separately. For the small *A. taeniatus*, the frequency of fin biting (per 30 min) on Ishigaki Island (*N*_1_) was significantly higher than that on Sesoko Island (*N*_2_) (medians = 6 and 1.33, ranges = 3–22 and 0–8, respectively; *N*_1_ = 5, *N*_2_ = 19; Mann–Whitney *U* test: *U* = 7.5, *P* = 0.004; Fig. 4a). For the large *A. taeniatus*, however, the frequency of fin biting was not significantly different between the two locations (medians = 0 and 0.25, ranges = 0 and 0–1.5, respectively; *N*_1_ = 3, *N*_2_ = 21; Mann–Whitney *U* test: *U* = 13.5, *P* = 0.09; Fig. 4b); fin biting was not observed in the large *A. taeniatus* on Ishigaki Island, partly because of the small sample size (*N*_1_ = 3).

**Figure 5:**
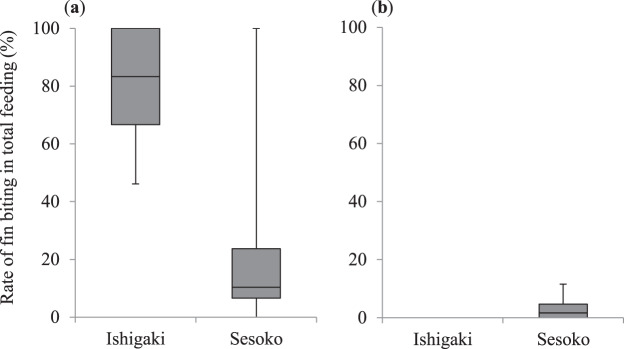
Comparison of the rate of fish-fin biting in the total feeding (%) by the false cleanerfish *Aspidontus taeniatus* between Ishigaki Island and Sesoko Island. (**a**) Small *A. taeniatus* (<7 cm total length [TL]), (**b**) large *A. taeniatus* (≥ 7 cm TL). Upper bars represent maximum, lower bars minimum, and middle bars median.

For the small *A. taeniatus*, the percentage of fin biting in the total feeding on Ishigaki Island (*N*_1_) was significantly higher than that on Sesoko Island (*N*_2_) (medians = 83% and 10%, ranges = 46–100% and 0–100%, respectively; *N*_1_ = 5, *N*_2_ = 19; Mann–Whitney *U* test: *U* = 8, *P* = 0.005; Fig. 5a). In contrast, for the large *A. taeniatus*, the percentage of fin biting in the total feeding was not significantly different between the two locations (medians = 0% and 1.66%, ranges = 0% and 0–11.5%, respectively; *N*_1_ = 3, *N*_2_ = 21; Mann–Whitney *U* test: *U* = 13.5, *P* = 0.09; Fig. 5b).

**Figure 6:**
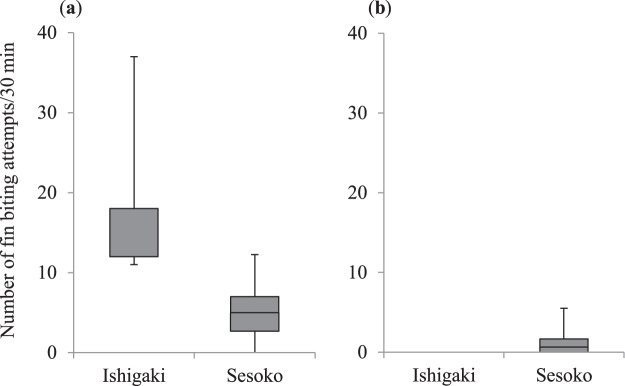
Comparison of the number of fish-fin biting attempts by the false cleanerfish *Aspidontus taeniatus* between Ishigaki Island and Sesoko Island. (**a**) Small *A. taeniatus* (<7 cm total length [TL]), (**b**) large *A. taeniatus* (≥ 7 cm TL). Upper bars represent maximum, lower bars minimum, and middle bars median.

The number of attempts (per 30 min) for biting fish fins (i.e., the number of approaches to the target fish) on Ishigaki Island (*N*_1_) was also significantly higher than that on Sesoko Island (*N*_2_) for the small *A. taeniatus* (medians = 12 and 5, ranges = 11–37 and 0–12.3, respectively; *N*_1_ = 5, *N*_2_ = 19; Mann–Whitney *U* test: *U* = 3, *P* = 0.002; Fig. 6a), but was not significantly different between the two locations for the large *A. taeniatus* (medians = 0 and 0.6, ranges = 0 and 0–5.5, respectively; *N*_1_ = 3, *N*_2_ = 21; Mann–Whitney *U* test: *U* = 10.5, *P* = 0.06; Fig. 6b).

**Figure 7:**
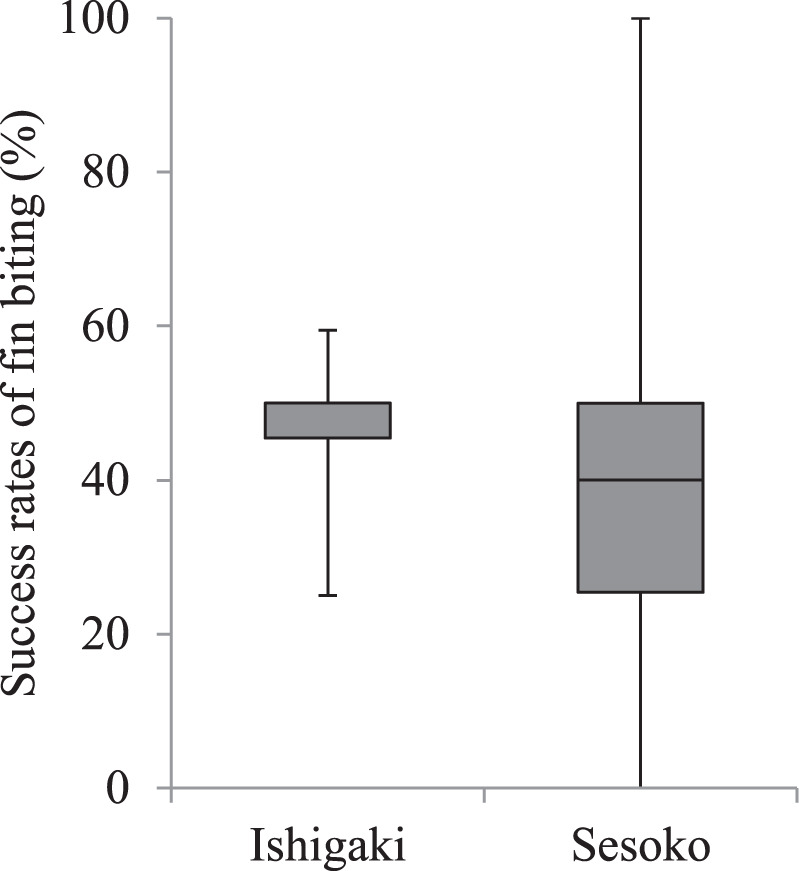
Comparison of the success rate of fish-fin biting (%) by the false cleanerfish *Aspidontus taeniatus* of the small size class (<7 cm total length) between Ishigaki Island and Sesoko Island. Upper bars represent maximum, lower bars minimum, and middle bars median.

The success rate of fin biting was not significantly different between the two locations for the small *A. taeniatus* (medians = 50% and 40%, ranges = 25–59% and 0–100%, respectively; *N*_1_ = 5, *N*_2_ = 18; Mann–Whitney *U* test: *U* = 34, *P* = 0.41; Fig. 7). We could not compare the success rate across the groups of large *A. taeniatus* because they did not display fin biting on Ishigaki Island.

## Discussion

Our study revealed that the abundance of other food items underlies the geographical variation in aggressive mimicry behaviour (i.e. the frequency of fish-fin biting) by *A. taeniatus*. The frequency of fin biting for the small *A. taeniatus* on Ishigaki Island, where *S. giganteus* and *T. crocea* were very rare, was significantly higher than that on Sesoko Island, where the two food items were abundant^21^. Therefore, we suggest that the high frequency of fin biting in French Polynesia relative to other localities^20^ may also be the result of scarce benthic food items, although their abundance was not studied.

In contrast to the small *A. taeniatus*, there was no significant difference in the frequency of fin biting for the large *A. taeniatus* between Ishigaki Island and Sesoko Island. Fujisawa *et al*.^21^ reported that the *A. taeniatus* bites fish fins more frequently on Sesoko Island when it is small. We found a similar tendency on Ishigaki Island in this study. It was also reported that a high frequency of fin biting was only observed in juvenile *A. taeniatus* in Indonesia^20^. Thus, it is suggested that the frequency of fish-fin biting decreases with growth in any location. However, the frequency of fish egg eating increased with growth on Sesoko Island^21^. We could not detect such a relationship on Ishigaki Island: egg eating was only observed once, probably due to the small sample size (*N* = 3) of the large *A. taeniatus*.

The feeding frequencies on *S. giganteus* and *T. crocea* on Ishigaki Island were much lower than those on Sesoko Island, simply because these benthic food items were very rare on Ishigaki Island. In contrast, the small *A. taeniatus* bit fish fins more frequently on Ishigaki Island than on Sesoko Island, although the number of fishes targeted by fin biting was not different between the two locations. The small *A. taeniatus* on Ishigaki Island had to rely on fin biting because they could not utilise *S. giganteus* and *T. crocea*. Although Cheney *et al*.^20^ suggested that the frequency of fish-fin biting may be related to the abundance of other food items, this was tested and confirmed for the first time in this study.

In the small *A. taeniatus*, not only the frequency of fin biting, but also the percentage of fin biting in the total feeding on Ishigaki Island, was higher than that on Sesoko Island. Although the success rate of fin biting was not different between the two locations, the number of fin biting attempts was significantly higher on Ishigaki Island. These results support the conclusion that the small *A. taeniatus* on Ishigaki Island had to rely on fin biting, although the success rate was not high. Thus, small *A. taeniatus* strongly rely on aggressive mimicry when other food items are rare. Although Kuwamura^17^ suggested that the principal function of this mimicry is not aggressive mimicry but immunity from predation (protective mimicry), we conclude that aggressive mimicry is important for the survival of small *A. taeniatus*, especially when benthic foods are rare in their local habitat.

Large *A. taeniatus* need not rely on aggressive mimicry (fin biting) if they can utilise fish eggs, *S. giganteus* and *T. crocea*^21^. Since the sample size of the large *A. taeniatus* on Ishigaki Island was small in this study, further observations are needed, with a focus on the feeding tactics of the larger *A. taeniatus* when food items other than fish fin are rare, to examine whether they can also rely on aggressive mimicry in such conditions. Moreover, although it has been suggested that this mimicry serves a protective function^1,17,19–21^, no experimental data have been reported to test this function, and further studies are needed.

## Methods

To contrast the findings from Sesoko Island in the Okinawa Islands^21^, field observational surveys were conducted during August 2017 on four fringing reefs along the north coast of Ishigaki Island in the Yaeyama Islands, the southernmost area of Okinawa (ca. 440 km southwestward from Sesoko Island): Yonehara Beach (24°45′N, 124°18′E; approx. 170 × 220 m), Crystal Beach (24°45′N, 124°17′E; approx. 100 × 240 m), Yoshihara Beach (24°45′N, 124°16′E; approx. 380 × 610 m), and Kabira-ishizaki Beach (24°48′N, 124°11′E; approx. 80 × 460 m). When we found *A. taeniatus*, we tracked it for 30 min by snorkelling. Its population density was very low; only nine individuals (5–11 cm TL) were found (six at Yonehara, none at Crystal Beach, two at Yoshihara, and one at Kabira-ishizaki), and 30-min observations were made between one and three times (420 min in total) for eight of them, except for the largest one. We recognised individuals based on differences in body size and colour patterns confirmed in the photos. All of them showed body colour similar to the adult, not juvenile, of *L. dimidiatus*. We estimated the TL of the observed fish at each 1 cm size class (e.g., size class 6 cm TL = 60–69 mm TL).

We recorded the feeding behaviours of *A. taeniatus* according to the methods of a previous study^21^ conducted on the reefs of Sesoko Island. We recorded their behaviour with a waterproof datasheet and sometimes took photographs and video using an underwater digital camera (TG-3 Tough; Olympus, Tokyo, Japan). For the feeding behaviour of *A. taeniatus*, food-related items were noted as (i) biting fins of other fishes, (ii) predation on demersal fish eggs, (iii) biting body parts of benthic animals, and (iv) others^21^. We recorded the number of fin-biting attempts (i.e., “trials” in Fujisawa *et al*.^21^) that succeeded and failed. We directly counted nipping behaviour on eggs when *A. taeniatus* intruded into the open nests of damselfishes, but when *A. taeniatus* intruded into closed-type nests established in holes or crevices, we estimated nipping numbers using the frequency data taken from the open-type nests (i.e. average 10 times per 30 s)^21^. In cases of predation on benthic animals, we recorded the target species name and the number of bites.

To evaluate the amount of food items, we counted them every 1 × 5 m within the home range of each focal individual of *A. taeniatus* after its 30 min observation (e.g. if the home range was 10 m wide, two 1 × 5 m line censuses were performed) and analysed the average number per 5 m^2^. The counted food items were *S. giganteus*, *T. crocea*, and target fish for fin biting^21^. Demersal fish eggs were not counted because it was difficult to find them in closed-type nests. Since the line census was not conducted off Sesoko Island when the behavioural data were collected in 2016^21^, seven 1 × 5 m line censuses were conducted at random in the 2016 study area during August 2017.

When comparing the data for Ishigaki Island (see Supplementary Table S1) with those for Sesoko Island, we decided to use the data collected by Fujisawa *et al*.^21^ (see Supplementary Table S2), except for the data for the above-mentioned line censuses (see Supplementary Table S3). We used nonparametric tests for statistical analyses because of the non-normality and heterogeneous variances within the data. Therefore, we used a median and range combination as an indicator of the data characteristics. To examine the relationship between *A. taeniatus* body size and the frequency of feeding on each item, we used Spearman’s rank correlation test. We used the Mann–Whitney *U* test to analyse the differences between Ishigaki Island and Sesoko Island, and those between the small and large size classes of *A. taeniatus*. Statistical analyses were conducted using R ver. 3.5.2 software (R Development Core Team, 2018)^22^.

### Ethical statement

We observed feeding behaviour of a coral reef fish, *Aspidontus taeniatus*, by snorkeling in nature without catching and tagging the target fish. Thus, no ethic or law violations are included in the present study. All procedures performed in this study were in accordance with the Guidelines for the Proper Conduct of Animal Experiments and related activities laid down by the Hiroshima University Animal Research Committee (No. 020A170410 certificated on April 10th, 2017), the ASAB/ABS Guidelines for the Use of Animals in Research (Guidelines for the treatment of animals in behavioural research and teaching; 10.1016/j.anbehav.2019.11.002), the Guidelines for the Use of Fishes in Research by the Ichthyological Society of Japan (http://www.fish-isj.jp/english/guidelines.html), and the Guideline for Ethological Studies by the Japan Ethological Society (http://www.ethology.jp/guideline.pdf).

## Supplementary information

Supplementary information.

## Data Availability

All data generated or analysed during this study are included in the Supplementary Information files.
