# Supplementary material for: The false cleanerfish relies on aggressive mimicry to bite fish fins when benthic foods are scarce in their local habitat
Source: Sci Rep. 2020 May 26;10:8652. doi: 10.1038/s41598-020-65304-6 (PMC7250849; doi:10.1038/s41598-020-65304-6)
Supplement: Supplementary file 1 — Supplementary information. [file 41598_2020_65304_MOESM1_ESM.docx]

**Supplementary Information**

The false cleanerfish relies on aggressive mimicry to bite fish fins when benthic foods are scarce in their local habitat

Misaki Fujisawa, Yoichi Sakai, Tetsuo Kuwamura

For the benthic food items in the following tables: tubeworm, *Spirobranchus giganteus*; boring clam, *Tridacna crocea.*

Table S1 Feeding frequency on each food item and the number of fish-fin biting attempts for each individual of the false cleanerfish *Aspidontus taeniatus* on Ishigaki Island (*N* = 8 individuals)

|  |  |  |  |  |  |  |  |  |  |
| --- | --- | --- | --- | --- | --- | --- | --- | --- | --- |
|  |  |  |  |  |  |  |  |  |  |
|  | Total length (cm) | Number of feedings/30 min | | | | |  | Number of  fin biting attempts/30 min |  |
|  |  | Tubeworm | Boring clam | Fin of  other fishes | Demersal fish eggs | Others |  |  |  |
|  | 5 | 0.0 | 0.0 | 22.0 | 0.0 | 0.0 |  | 37.0 |  |
|  | 6 | 1.0 | 0.0 | 6.0 | 0.0 | 2.0 |  | 11.0 |  |
|  | 6 | 3.5 | 0.0 | 3.0 | 0.0 | 0.0 |  | 12.0 |  |
|  | 6 | 0.0 | 0.0 | 9.0 | 0.0 | 0.0 |  | 18.0 |  |
|  | 6 | 0.0 | 1.0 | 5.0 | 0.0 | 0.0 |  | 11.0 |  |
|  | 8 | 0.0 | 0.0 | 0.0 | 0.0 | 4.5 |  | 0.0 |  |
|  | 9 | 0.0 | 0.0 | 0.0 | 0.0 | 4.0 |  | 0.0 |  |
|  | 10 | 0.3 | 0.0 | 0.0 | 1.7 | 3.0 |  | 0.0 |  |
|  |  |  |  |  |  |  |  |  |  |
|  |  |  |  |  |  |  |  |  |  |

Table S2 Feeding frequency on each food item and the number of fish-fin biting attempts for each individual of the false cleanerfish *Aspidontus taeniatus* on Sesoko Island (*N* = 40 individuals: the same data set used in [21])

|  | |  |  |  |  |  |  |  |  |  |
| --- | --- | --- | --- | --- | --- | --- | --- | --- | --- | --- |
|  | Total length (cm) | | Number of feedings/30 min | | | | |  | Number of  fin biting attempts/30 min |  |
|  |  |  | Tubeworm | Boring clam | Fin of  other fishes | Demersal fish eggs | Others |  |  |  |
|  | 5 | | 13.2 | 0.6 | 3.2 | 0.0 | 0.4 |  | 6.6 |  |
|  | 5 | | 0.0 | 0.0 | 2.0 | 0.0 | 0.0 |  | 7.0 |  |
|  | 5 | | 17.0 | 1.0 | 1.0 | 0.0 | 0.0 |  | 5.0 |  |
|  | 5 | | 7.0 | 19.0 | 3.0 | 0.0 | 0.0 |  | 7.0 |  |
|  | 5 | | 5.0 | 7.0 | 1.0 | 0.0 | 0.0 |  | 7.0 |  |
|  | 5 | | 5.7 | 2.3 | 4.3 | 0.0 | 0.0 |  | 7.0 |  |
|  | 6 | | 4.6 | 0.6 | 1.0 | 3.8 | 0.8 |  | 1.0 |  |
|  | 6 | | 0.0 | 1.0 | 0.0 | 0.0 | 0.0 |  | 0.0 |  |
|  | 6 | | 8.0 | 6.5 | 4.0 | 0.0 | 2.8 |  | 6.3 |  |
|  | 6 | | 10.3 | 0.0 | 8.0 | 0.0 | 1.8 |  | 12.3 |  |
|  | 6 | | 20.0 | 2.3 | 1.3 | 0.0 | 0.7 |  | 2.7 |  |
|  | 6 | | 6.5 | 6.5 | 0.5 | 0.0 | 0.0 |  | 1.5 |  |
|  | 6 | | 9.3 | 0.3 | 1.1 | 0.3 | 0.6 |  | 4.6 |  |
|  | 6 | | 6.0 | 3.0 | 1.3 | 0.0 | 0.0 |  | 2.7 |  |
|  | 6 | | 0.0 | 0.0 | 4.0 | 0.0 | 0.0 |  | 10.0 |  |
|  | 6 | | 3.0 | 2.0 | 2.0 | 0.0 | 0.0 |  | 5.0 |  |
|  | 6 | | 1.5 | 1.5 | 0.5 | 0.0 | 2.0 |  | 4.0 |  |
|  | 6 | | 7.8 | 3.0 | 0.0 | 0.6 | 0.8 |  | 0.2 |  |
|  | 6 | | 2.5 | 6.5 | 1.5 | 0.0 | 1.5 |  | 4.3 |  |
|  | 7 | | 5.0 | 2.0 | 1.0 | 0.7 | 0.0 |  | 1.7 |  |
|  | 7 | | 4.7 | 11.7 | 0.7 | 0.0 | 1.0 |  | 1.7 |  |
|  | 7 | | 1.0 | 11.5 | 1.5 | 0.0 | 0.0 |  | 5.5 |  |
|  | 7 | | 11.0 | 0.0 | 0.0 | 0.0 | 0.0 |  | 0.0 |  |
|  | 7 | | 7.0 | 5.0 | 0.0 | 0.0 | 0.0 |  | 0.0 |  |
|  | 7 | | 21.0 | 0.5 | 1.5 | 0.0 | 0.0 |  | 4.5 |  |
|  | 7 | | 9.0 | 3.0 | 0.0 | 0.0 | 3.5 |  | 1.5 |  |
|  | 8 | | 2.0 | 0.0 | 0.2 | 1.7 | 0.3 |  | 0.3 |  |
|  | 8 | | 6.0 | 12.5 | 1.0 | 0.0 | 0.0 |  | 2.0 |  |
|  | 8 | | 4.5 | 10.0 | 0.3 | 0.0 | 0.3 |  | 0.8 |  |
|  | 8 | | 0.7 | 0.7 | 0.3 | 4.7 | 3.0 |  | 0.3 |  |
|  | 8 | | 6.7 | 0.0 | 0.0 | 4.0 | 0.0 |  | 0.0 |  |
|  | 9 | | 20.0 | 0.5 | 1.0 | 0.0 | 0.0 |  | 2.0 |  |
|  | 9 | | 0.2 | 0.0 | 0.0 | 0.0 | 10.6 |  | 0.0 |  |
|  | 9 | | 4.0 | 0.0 | 0.0 | 0.0 | 0.5 |  | 0.0 |  |
|  | 9 | | 8.0 | 3.5 | 0.5 | 0.5 | 0.0 |  | 1.0 |  |
|  | 10 | | 1.4 | 0.1 | 0.0 | 2.9 | 0.5 |  | 0.0 |  |
|  | 10 | | 4.0 | 0.0 | 0.0 | 7.0 | 0.0 |  | 1.0 |  |
|  | 10 | | 15.6 | 1.0 | 0.3 | 2.4 | 0.4 |  | 0.6 |  |
|  | 10 | | 0.0 | 0.0 | 0.0 | 5.5 | 2.0 |  | 0.0 |  |
|  | 10 | | 3.5 | 4.0 | 0.5 | 0.0 | 0.0 |  | 0.5 |  |
|  |  | |  |  |  |  |  |  |  |  |

Table S3 Density (per 5 m^2^) of each food item of the false cleanerfish *Aspidontus taeniatus* on Ishigaki Island (*N* = 7 individuals) and Sesoko Island (*N* = 7 sites)

|  |  |  |  |  |  |  |  |  |
| --- | --- | --- | --- | --- | --- | --- | --- | --- |
|  | Ishigaki Island | | |  | Sesoko Island | | |  |
|  | Tubeworm | Boring clam | Target fish |  | Tubeworm | Boring clam | Target fish |  |
|  | 0.2 | 0.0 | 36.1 |  | 2.0 | 2.5 | 42.0 |  |
|  | 0.0 | 0.0 | 41.0 |  | 2.0 | 3.0 | 61.0 |  |
|  | 0.6 | 0.0 | 29.8 |  | 0.0 | 45.0 | 18.0 |  |
|  | 0.0 | 0.0 | 44.0 |  | 0.8 | 1.5 | 7.5 |  |
|  | 0.0 | 0.0 | 52.0 |  | 15.0 | 8.0 | 51.0 |  |
|  | 0.0 | 2.0 | 17.0 |  | 11.0 | 5.0 | 37.0 |  |
|  | 0.0 | 0.0 | 37.0 |  | 3.3 | 36.7 | 4.0 |  |
|  |  |  |  |  |  |  |  |  |
